# Morpho-histological characterisation of the alimentary canal of an important food fish, Asian seabass (*Lates calcarifer*)

**DOI:** 10.7717/peerj.2377

**Published:** 2016-08-24

**Authors:** Kathiresan Purushothaman, Doreen Lau, Jolly M. Saju, Syed Musthaq SK, Declan Patrick Lunny, Shubha Vij, László Orbán

**Affiliations:** 1 Reproductive Genomics Group, Temasek Life Sciences Laboratory, Singapore, Singapore; 2 Institute of Medical Biology, Agency for Science, Research and Technology, Singapore; 3 Centre for Comparative Genomics, Murdoch University, Murdoch, Australia; 4 Department of Animal Sciences and Animal Husbandry, Georgikon Faculty, University of Pannonia, Keszthely, Hungary

**Keywords:** Gut, *Lates calcarifer*, Morphohistology, Fish alimentary canal

## Abstract

Asian seabass (*Lates calcarifer*) is a food fish of increasing aquaculture importance. In order to improve our understanding on the digestive system and feeding of this species, morphological and histological features of the gut were studied. Morphologically, the Asian seabass gut is defined by a short and muscular esophagus, well-developed stomach and comparatively short intestine. Mucous secreting goblet cells reactive to PAS (Periodic Acid Schiff) and AB (Alcian Blue) stain were present throughout the esophagus. The stomach was sac-like and could be distinguished into the cardiac, fundic and pyloric regions. Gastric glands and mucus cells were predominately present in the cardiac and fundic regions. Five finger-like pyloric caeca were present between the stomach and intestine. The intestine was a short, tubular structure with no morphological differences between the various regions. Histologically, the intestinal regions were similar, the main difference being in the number of goblet cells that increased from anterior to posterior intestine, with 114 ± 9, 153 ± 7 and 317 ± 21 goblet cells in the anterior, mid and posterior regions, respectively. The intestinal epithelium stained positively for PAS, but the staining was stronger for acidic glycoproteins. The rectum was similar to intestine, except for increased goblet cell numbers (anterior rectum: 529 ± 26; posterior rectum: 745 ± 29). Gut morpho-histology did not respond to salinity changes, however, there was a significant reduction of mucosal height, goblet cell numbers and muscularis thickness upon food deprivation.

## Introduction

The Asian seabass (*Lates calcarifer*; *Centropomidae*), also known as barramundi or giant perch, is an important food fish in many parts of the world (Nelson, 2006; Vij et al., 2016; Vij et al., 2014). As such, there has been an increasing interest in its growth mechanism and nutritional needs in recent years, especially with a view to improve the production of good fillet of marketable quantity and quality (Alhazzaa et al., 2011; Glencross et al., 2008; Katersky & Carter, 2009; Matthews et al., 1997; Ngoh et al., 2015; Srichanun et al., 2013; Tu et al., 2013; Xia et al., 2013).

The Asian seabass is an opportunistic predator that naturally feeds on live crustaceans, molluscs and pelagic bony fishes (Davis, 1985). In commercial aquaculture, however, the fishes are generally fed with frozen bait fish and pelleted feed composed of both plant and animal contents (Glencross, Wade & Morton, 2013).They exhibit moderate cannibalism, and may prey on their smaller siblings (Davis, 1985).The alimentary canal is one of the major organ systems of fishes that interacts with the environment. It plays a critical role in growth, nutrition, as well as survival of the fish under different conditions. A typical teleost gut is a tube-like structure beginning at the mouth and ending in the anus. The mouth serves to capture and sometimes, pre-process the food before it enters the esophagus. The latter is a short and muscular, mucous-secreting connection leading to the stomach. The stomach serves the purpose of digesting food, a function which is completed in the intestine. The intestine is the main organ for nutrient absorption, usually aided by the variable finger-like appendages, the pyloric caeca, present between the stomach and intestine which serve to increase the surface area of the intestine. The undigested food is expelled through the anus, aided by the many mucous secreting cells located in the posterior intestine and rectum. The alimentary canal shows distinct differences in terms of morphology and function in relation to the type of food and feeding habits, as well as body weight and sex (Day, Tibbetts & Secor, 2014; Gu et al., 2014)

Over the past two decades, an increasing number of research projects and publications have been focusing on the Asian seabass (Kuznetsova et al., 2014; Ngoh et al., 2015). Selection projects utilized the tools of molecular genetics to improve seabass lines (Wang et al., 2011; Wang et al., 2006), transcriptomic studies have analyzed the genetic regulation of its sex change (Ravi et al., 2014) or responses to a vaccine and bacterial infection (Jiang et al., 2014) and others have investigated the evolution of the species (Vij et al., 2016; Vij et al., 2014). One of the areas of primary focus was to study the effects of feeds onto the fish using molecular tools (Ngoh et al., 2015). A good understanding of the functional morphology of the Asian seabass alimentary canal is fundamental for learning more about their feeding physiology and habits especially for feed formulation prior to production age. Furthermore, studies related to the growth and nutritional needs of the species have been focussed on feed formulation trials (Alhazzaa et al., 2011; Boonyaratpalin & Williams, 2001; Tu et al., 2013). Studies have also been performed to improve our understanding of the feeding dynamics of Asian seabass based on biochemical analysis of digestive enzymes in the larval/juvenile stages (Monjoyo, Tan-Fermin & Macaranas, 2003; Walford & Lam, 1993) and stomach content analysis of the adult fish (Davis, 1985). However, there is limited information available on the functional characterisation and morphological analysis of the Asian seabass alimentary canal and that information is essential for better understanding the potential effects of feeds onto the digestive system of the fish.

According to our knowledge, this is the first report on the morphological and histological analysis of digestive system of Asian seabass. We performed a detailed examination of the alimentary canal of this economically important food fish species and describe our findings in relation to its feeding behaviour and environment. The structural features of the gut correlated well with the feeding habits of a carnivorous fish species. In order to identify the components of mucus substances secreted from the mucus cells present in the various regions of the gut, we used Periodic acid-Schiff (PAS) and Alcian Blue (AB) staining for the joint detection of neutral and acid glycoproteins. The knowledge gained here will be utilized for setting up a reference/baseline for the healthy seabass gut, which would be useful for understanding changes to the digestive system under altered conditions such as disease or varied feed/feeding regime. This, in turn will help the development of an informed aquaculture program and further improve the management of Asian seabass stocks.

## Materials and Methods

### Ethics statement

Farmed Asian seabass were obtained from the Marine Aquaculture Centre (Singapore). All experiments were approved by Agri-food and Veterinary Authority (AVA) Institutional Animal Care and Use Committee (IACUC) (approval ID: AVA-MAC-2012-02) and performed according to guidelines set by the National Advisory Committee on Laboratory Animal Research (NACLAR) for the care and use of animals for scientific research in Singapore.

### Sample collection

For morpho-histological analyses, three-month-old Asian seabass fed with commercial feed were used for studying the morpho-histology. Samples had a mean weight of 71.3 ± 20.7 g, standard length of 13.2 ± 1.2 cm and total length 16.3 ± 1.5 cm at the time of sacrifice. The height of mucosal folds from different regions of the Asian seabass gut was also measured.

For measuring the intestinal coefficient, we procured twenty three, three-month-old Asian seabass from a commercial farm based in Singapore. These fishes had a mean standard length (SL) of 7.7 ± 0.86 cm and a mean body weight (BW) of 15.3 ± 3.3 g. The fishes were dissected to obtain the gastrointestinal tract and from which the length of intestine (IL; cm) was measured. The intestinal coefficient (IC) was calculated using the formula: IC = IL/SL as described earlier (Angelescu & Gneri, 1949).

For the starvation study, Asian seabass individuals fed with commercial feed until four months of age were starved for one and three weeks respectively, while the control group was continued to be fed commercial feed (thrice per day). In each case, three individuals from three separate tanks (a total of nine for each group—control and starvation) were collected for sampling both at the first as well as third week.

For the salinity stress experiment, ca. 300 Asian seabass individuals were reared in seawater (30–32 ppt) until 44 dph. Around 75 fishes were transferred to a separate tank at 30–32 ppt to serve as a control while ∼75 fishes were transferred to fresh water (0 ppt). The fishes in both control (30–32 ppt) and sample (0 ppt) tanks were kept for three weeks (i.e., till 65 dph). The remaining fishes from the control tank were allowed to grow for an additional 3 weeks under full seawater conditions, while the remaining fishes from the sample tank (0 ppt) were returned to seawater (30–32 ppt) at 65 dph and kept for 3 weeks (i.e., till 86 dph). From each group, the gut histology of at least six fishes was examined. A flow chart describing the experimental setup is given in Fig. S1.

In each case, the fishes were sacrificed by immersion in 2% tricaine. A ventral incision was performed to expose the coelomic cavity in order to remove organs of the alimentary canal for subsequent treatment and analysis.

### Histochemical preparation

For each individual, organ specimens from the alimentary canal were collected and fixed for 48 h in 10% buffered formalin for subsequent histological analyses. The fixed specimens were dehydrated using increasing concentrations of ethanol (50–100%) and embedded in hydroxyethyl methacrylate (Historesin, Leica). For each specimen, 10–20 cross sections (thickness: ∼5 µm) were obtained and mounted onto slides for subsequent histological staining by hematoxylin-eosin (HE). The sections were also stained for the identification of glycoproteins (Bancroft & Gamble, 2002). The combined staining of periodic acid-Schiff (PAS) and Alcian Blue (AB) (Novaultra staining kit, IHC World) was used for the joint detection of neutral and acid glycoproteins.

In addition, six histochemical procedures were used for the detection and visualization of different classes of glycoproteins (Table S2). Sections were stained with: (1) *α*-amylase digestion before PAS: identification of GPs with oxidizable vicinal diols; (2) acetylation before PAS: to block the oxidation of the 1,2 glycol groups by the periodic acid; (3) acetylation—saponification—PAS: to restore the 1,2 glycol groups which reacts with the periodic acid; (4) AB pH 2.5 (Alcian Blue 8GX, Sigma): to demonstrate glycoprotein’s with carboxyl groups (sialic acid or urinic acid) and/or with O-sulphate esters; (5) AB pH 1.0: to demonstrate glycoprotein’s with O-sulphate esters and (6) AB pH 0.5: to demonstrate strongly sulphated glycoproteins (Díaz et al., 2010). The specimens were examined using Zeiss Axioplan 2 mounted with a Nikon digital camera DXM 1200F and Zeiss AxioImager Z.2 mounted with CoolCube1 camera (in each case, 3–6 out of the 10–20 sections were evaluated in detail). Goblet cells were counted from same-sized villi (intestine/rectum: six villi per region) of five representative fishes and the results are shown as an average (±standard deviation). For both the starvation and salinity experiments, three cross-sections per specimen (six individuals per group) were quantified for mucosal height and muscularis thickness.

### Quantification and statistical analyses

All measurements were performed using Fiji software (Schindelin et al., 2012). The number of goblet cells significantly different between the three different regions of intestine and two regions of rectum was determined by Student’s *t*-test and significant difference in values is indicated by * (*p* < 0.05) (Table 1). Student’s *t*-test was also used to compare values between the control and test sample for both salinity and starvation experiments and significant difference in mean is indicated by * (*p* < 0.05).

**Table 1:** Goblet cell counts from different regions of the Asian seabass gut showing increased numbers of goblet cells from the anterior intestine to posterior rectum (*n* = 9).

Organ	Region	Goblet cell number
Intestine	Anterior	114.2 ± 9.1
	Mid	152.6 ± 7.1^*^
	Posterior	317.4 ± 20.7^*^
Rectum	Anterior	529.2 ± 26.3^*^
	Posterior	744.6 ± 29.3^*^

**Notes.**

* Indicates *p*-value (<0.05) calculated using Student’s *t*-test.

## Results

**Figure 1:**
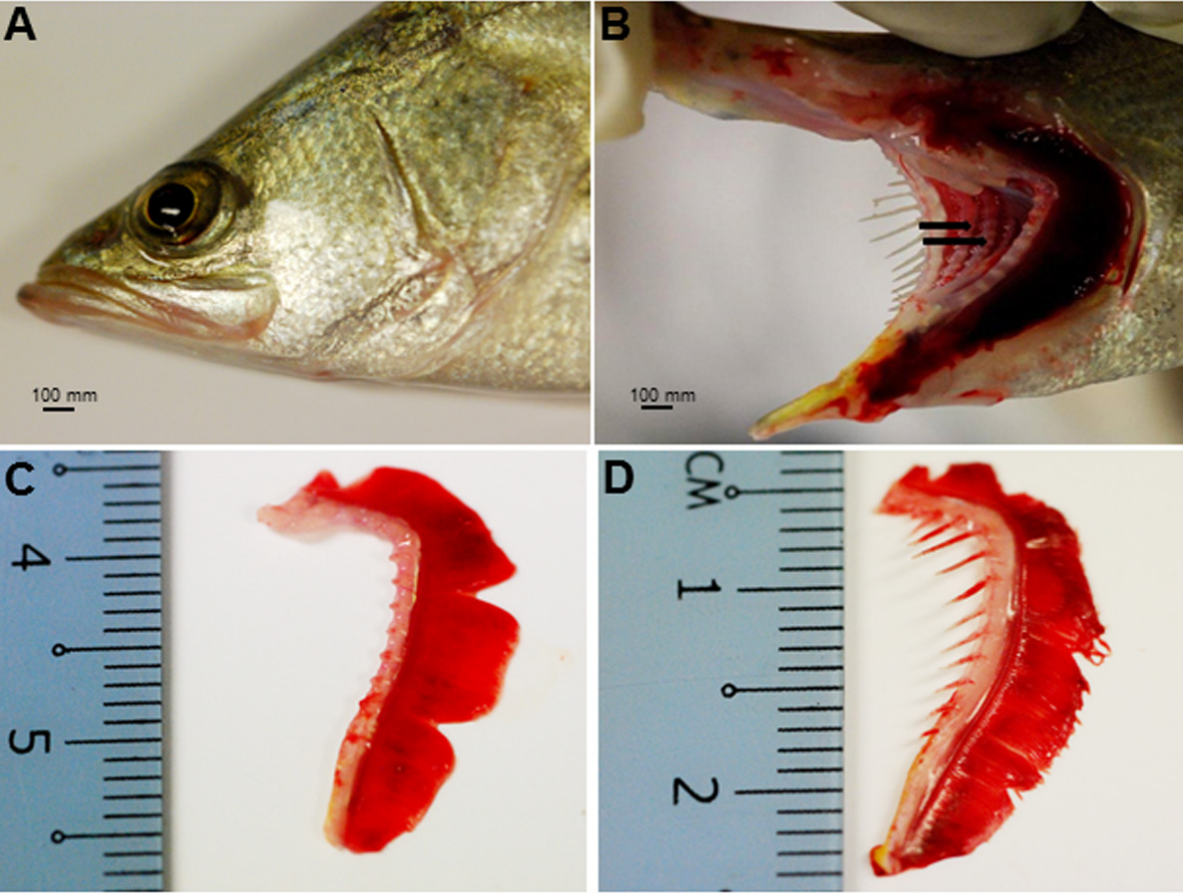
Gross morphology of the Asian seabass head. (A) Head portion of a three-month-old Asian seabass-mouth closed, (B) mouth opened to display the teeth (indicated by black arrows) and (C) the branchial arch without and (D) with gill rakers.

The Asian seabass showed an elongated and compressed body shape, with a large and slightly oblique mouth and upper jaw extended behind the eye (Fig. 1A). Three-month-old Asian seabass had a mean body weight (BW) of 15.3 ± 3.3 g, mean standard length (SL) of 7.7 ± 0.9 cm and mean intestinal length (IL) of 8.9 ± 0.9 cm. This yielded an intestinal coefficient (IC) of 1.1 ± 0.04, calculated using the formula: IC = IL/SL (Table S1). The teeth were villiform and were set in single rows on both sides of the jaw. No canine teeth could be seen (Fig. 1B). The gill chamber consisted of four gill arches, the first and second adorned with thin and long gill rakers, while the third and fourth gill arches lacked them (Figs. 1C–1D).

**Figure 2:**
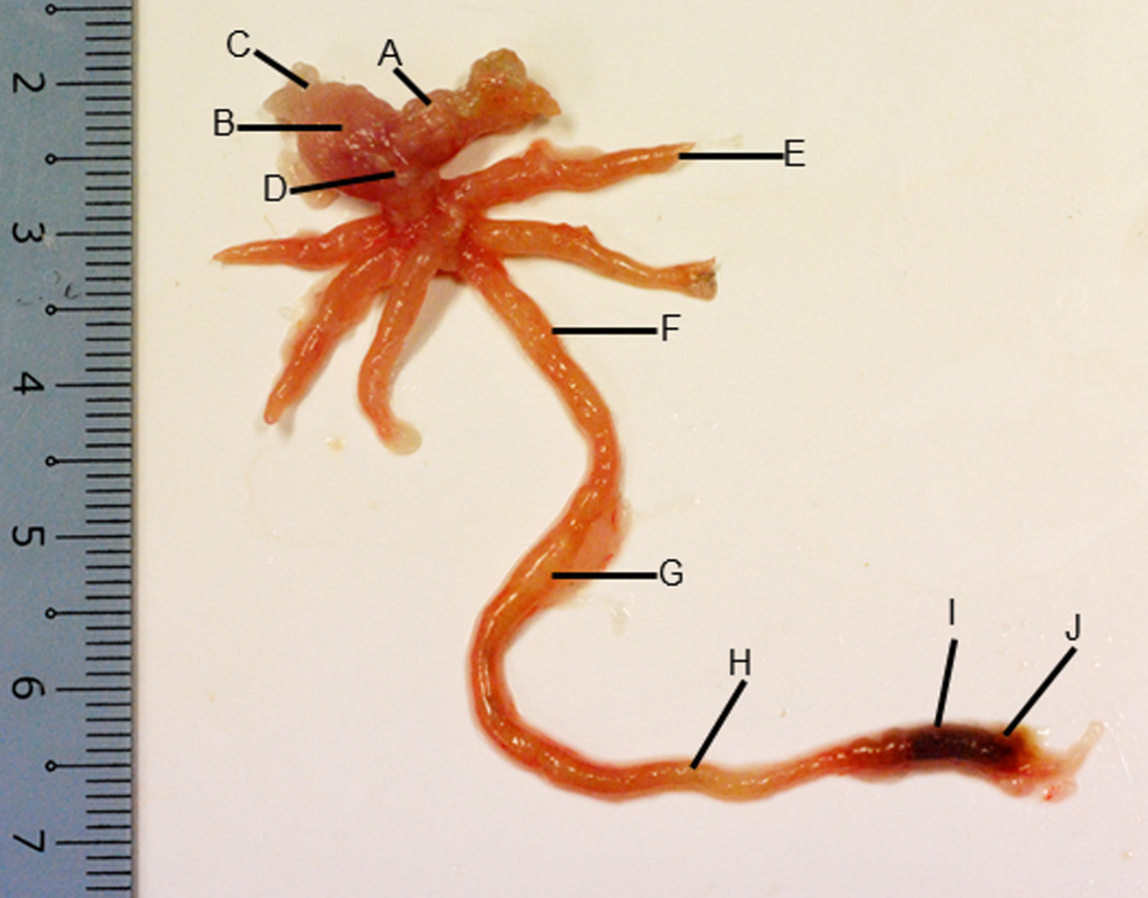
Gross morphology of the Asian seabass gut. (A) Esophagus, (B) cardiac stomach, (C) fundic stomach, (D) pyloric stomach, (E) pyloric caeca (F) anterior intestine, (G) mid intestine, (H) posterior intestine, (I) anterior rectum and (J) posterior rectum.

**Figure 3:**
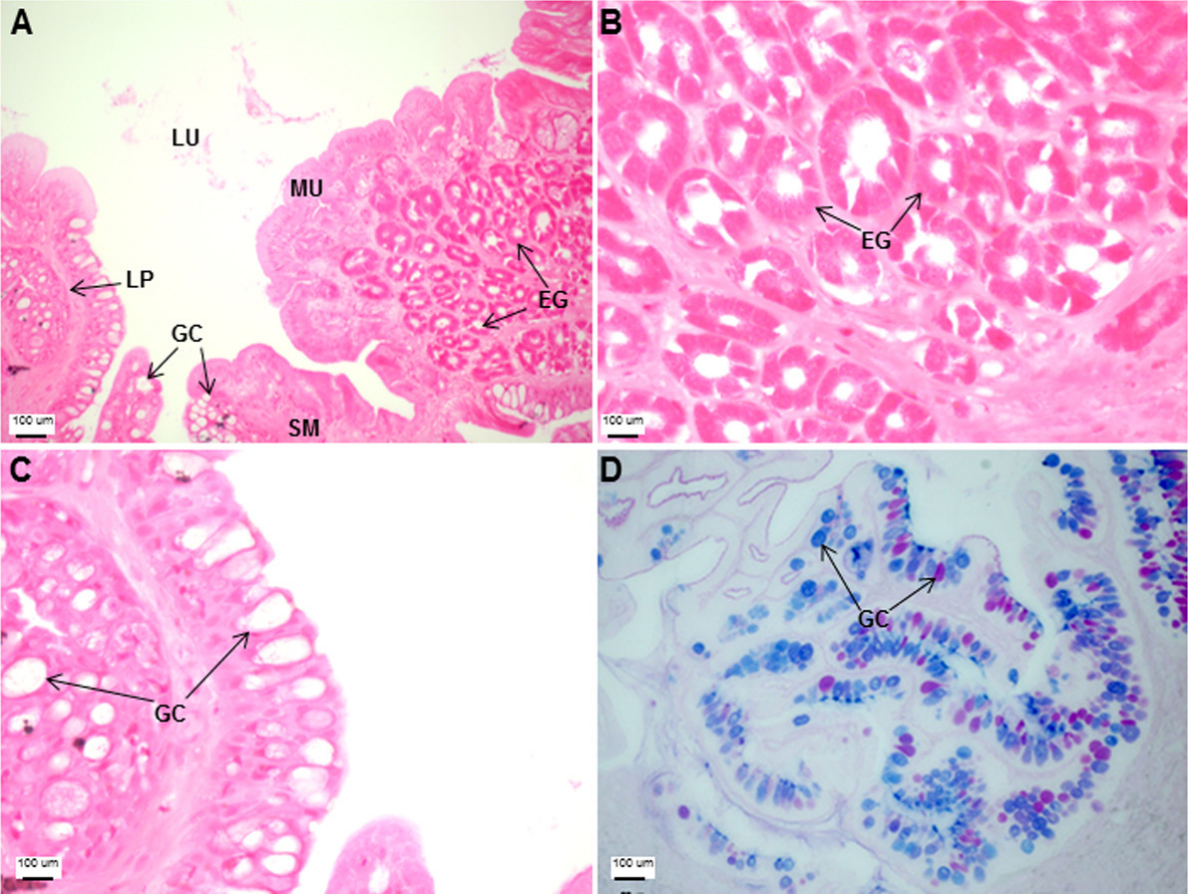
Both goblet cells and esophageal glands were detected in the Asian seabass esophagus by histological analysis. Transverse sections of the Asian seabass esophagus stained with (A: 10X; B and C: 63X) H&E and (D: 63X) PAS-AB pH 2.5. Abbreviations: lumen (LU), lamina propria (LP), mucosa (MU), submucosa (SM), esophageal glands (EG) and goblet cells (GC).

The esophagus was a short and thick-walled tubular structure, located between the end of the pharynx and anterior region of the cardiac stomach (Fig. 2A). It was comprised of four layers: mucosa, submucosa, muscularis and serosa (Figs. 3A–3C). The mucosa of the esophagus was a wrinkled mucosal epithelial fold, comprising of simple columnar epithelium with interspersed goblet cells and included the lamina propria, a layer packed with collagen fibres which extended towards the submucosa, a structure made up of connective tissues. The muscularis was present between the submucosa and serosa. It consisted of interweaving striated muscle fibers that extended towards the stomach and could be visualized as two distinct layers—the inner circular and outer longitudinal layers. Esophageal glands could also be seen in the mucosal region (Figs. 3A–3C). The majority of the goblet cells stained positively for both PAS and AB (Fig. 3D; Table S2) indicating the secretion of mucous containing neutral and acidic glycoproteins, respectively. However, there were few goblet cells which contained either only neutral or acidic glycoproteins.

**Figure 4:**
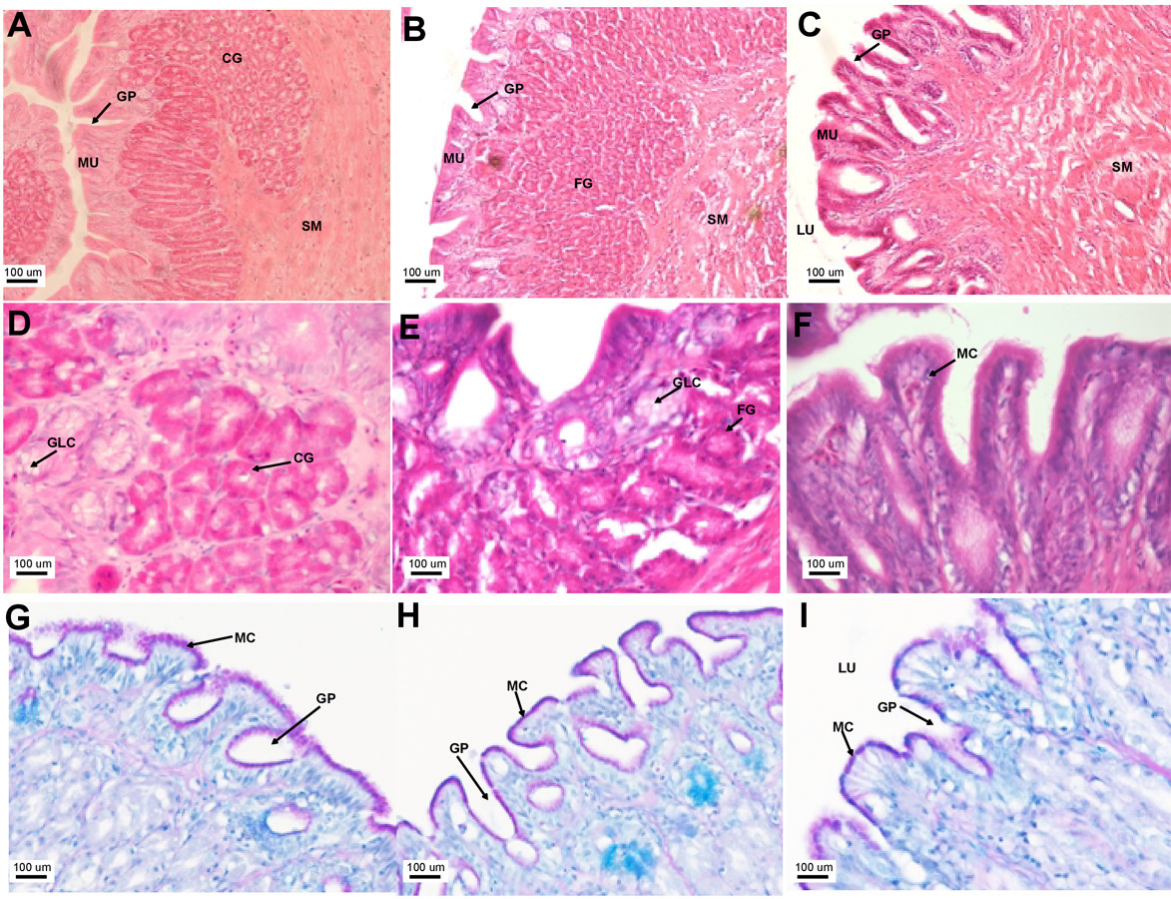
The Asian seabass stomach is divided into the cardiac, fundic and pyloric regions and shows the presence of glands and mucus cells. Transverse sections of the Asian seabass stomach stained with (A, B and C: 10X; D, E and F: 63X) H&E and (G, H and I: 63X) PAS-AB pH 2.5. The cardiac, fundic and pyloric regions are shown in (A, D, G)/(B, E, H) and (C, F, I), respectively. Abbreviations: Lumen (LU), gastric pits (GP), cardiac gland (CG), fundic gland (FG), lamina propria (LP), mucosa (MU), submucosa (SM), gland cells (GLC) and mucus secreting cells (MC).

**Figure 5:**
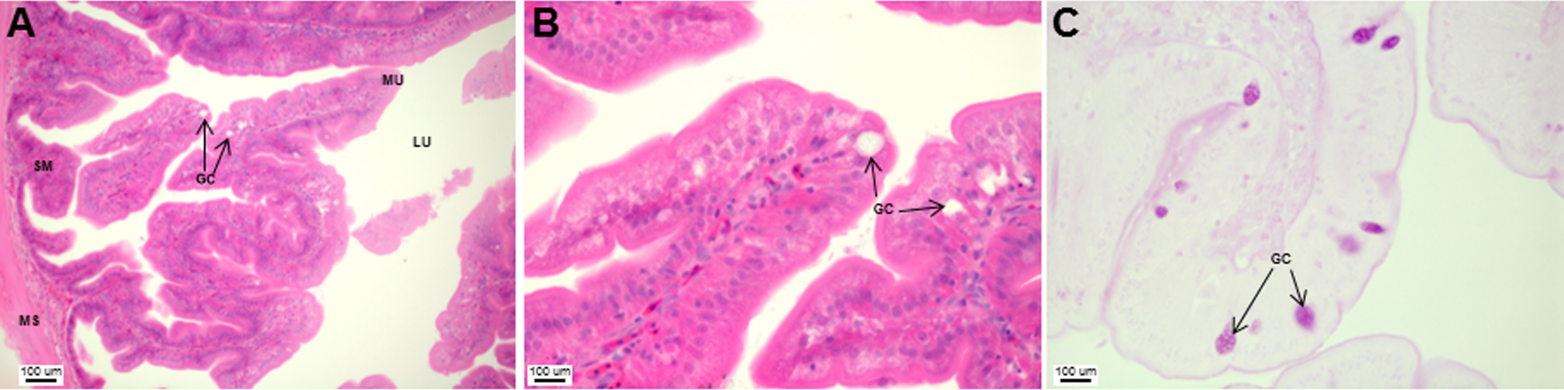
Asian seabass pyloric caeca resemble the intestine histologically, but with much fewer goblet cells. Transverse section of the pyloric caeca of Asian seabass stained with H&E (A and B; 10X and 63X, respectively) and PAS-AB pH 2.5 (C; 63X magnification). Abbreviations: lumen (LU), mucosa (MU), goblet cells (GC), submucosa (SM), and muscularis (MS).

**Figure 6:**
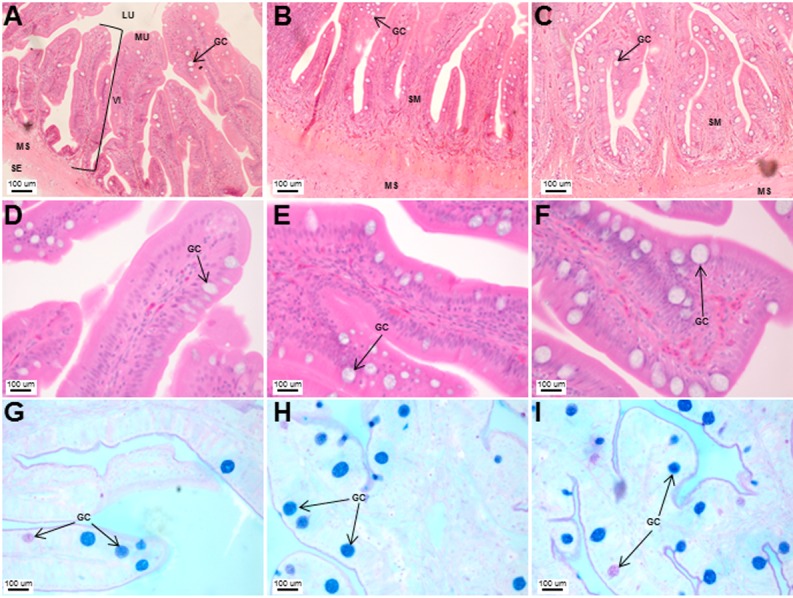
Histological analyses of the Asian seabass intestine did not reveal major differences from the anterior to posterior portion with the exception of goblet cell numbers. Transverse sections of the Asian seabass intestine stained with (A, B and C: 10X; D, E and F: 63X) H&E and (G, H and I: 63X) PAS-AB pH 2.5. The anterior, mid and posterior regions are shown in (A, D, G), (B, E, H) and (C, F, I), respectively. Abbreviations: Villi (VI), lumen (LU), mucosa (MU), submucosa (SM), muscularis (MS), goblet cells (GC) and serosa (SE).

**Figure 7:**
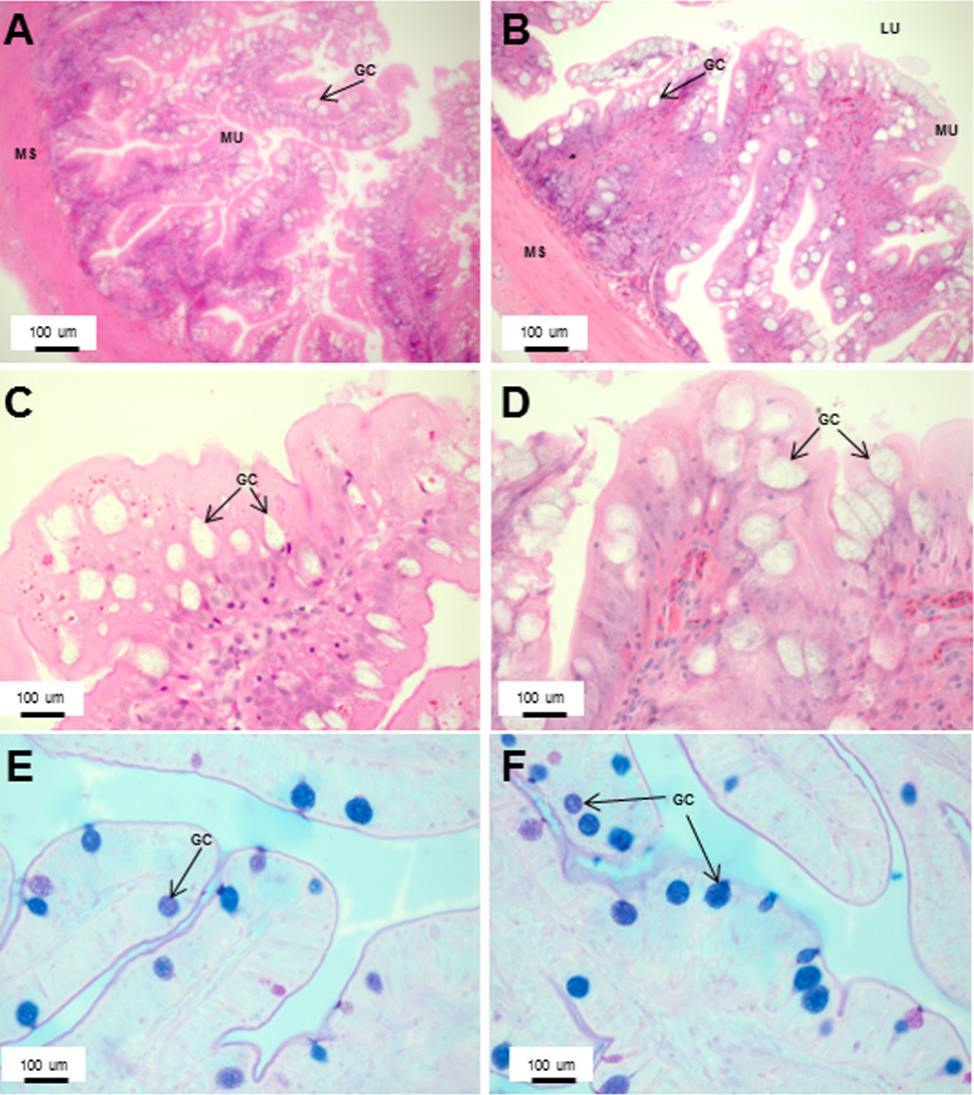
An increased number of goblet cells in the rectum compared to the intestine. Transverse sections of the Asian seabass rectum stained with (A and B: 10X; D and E: 63X) H&E and (E and F: 63X) PAS-AB pH 2.5. The anterior and posterior regions are shown in (A, C, E) and (B, D, F), respectively. Abbreviations: Villi (VI), lumen (LU), goblet cells (GC), mucosa (MU) and muscularis (MS).

The stomach was sac-like with the whole surface lined with secretory simple columnar epithelium and could be divided into three parts based on histology: the cardiac, fundic and pyloric regions (Figs. 2 and 4). Gastric pits (crypts) could be observed on the thick stomach mucosa with glands opening at the bottom of the pits which were most distinct in the pyloric region (Fig. 4). Both gastric glands and surface mucus secreting cells were predominately present in the cardiac and fundic regions of the stomach. The submucosa was seen as a loose connective tissue without the presence of glands. The surface mucus cells stained positive for PAS and only weakly for AB (pH 0.5/1.0/2.5), indicating the secretion of mucous containing neutral and to a lesser extent, acidic glycoproteins (Figs. 4G–4I; Table S2). The pyloric portion of the stomach (Fig. 4C) extended until the initial region of the intestinal tubes. The pyloric caeca consisted of five finger-like structures present between the connection of the stomach and anterior portion of the intestine (Fig. 2E). The mucosa with lengthy folds was comprised of a single layered epithelium with cylindrical goblet cells and lamina propria with loose connective tissue and small vessels (Fig. 5; mucosal fold length measurements from different regions of intestine are shown in Table S3). Absorptive cells, responsible for absorption of nutrients, were also observed in the epithelium. The villi were thinner and the number of goblets cells was lower in the pyloric caeca compared to the intestine. The pyloric caeca stained positive for PAS, but was negative for AB staining indicating the secretion of neutral glycoproteins (Table S2). The intestine of the Asian seabass was located between the pyloric caeca and rectum (Figs. 2F–2H). Though, there were no morphological differences, histologically, it could be divided into roughly three equal regions: the anterior, middle and posterior intestine (Figs. 6A–6F). The intestinal epithelium contained absorptive and goblet cells. The predominant type of goblet cells were stained for both AB and PAS, indicating the presence of both acidic and neutral glycoproteins, respectively (Figs. 6G–6I). In addition, a much lesser proportion of goblet cells which stained positive only for PAS or for AB could also be seen. The positive PAS staining disappeared following acetylation and could be retrieved after saponification (as also seen for rest of the alimentary tract) (Table S2). Glands could not be seen in either the mucosa or submucosa. The rectum (Figs. 2I–2J) extended from the posterior part of the intestine and ended ventrally at the anus in the facade of the anal fin. Similar to the intestine, the rectum contained mucosal folds without glands (Figs. 7A–7D). The number of goblet cells showed a progressive increase along the length of the intestinal tract with the posterior part of rectum having over six times the number than the anterior intestine (see Table 1 for the cell counts for each region; and Figs. 7C–7D for representative examples).

**Figure 8:**
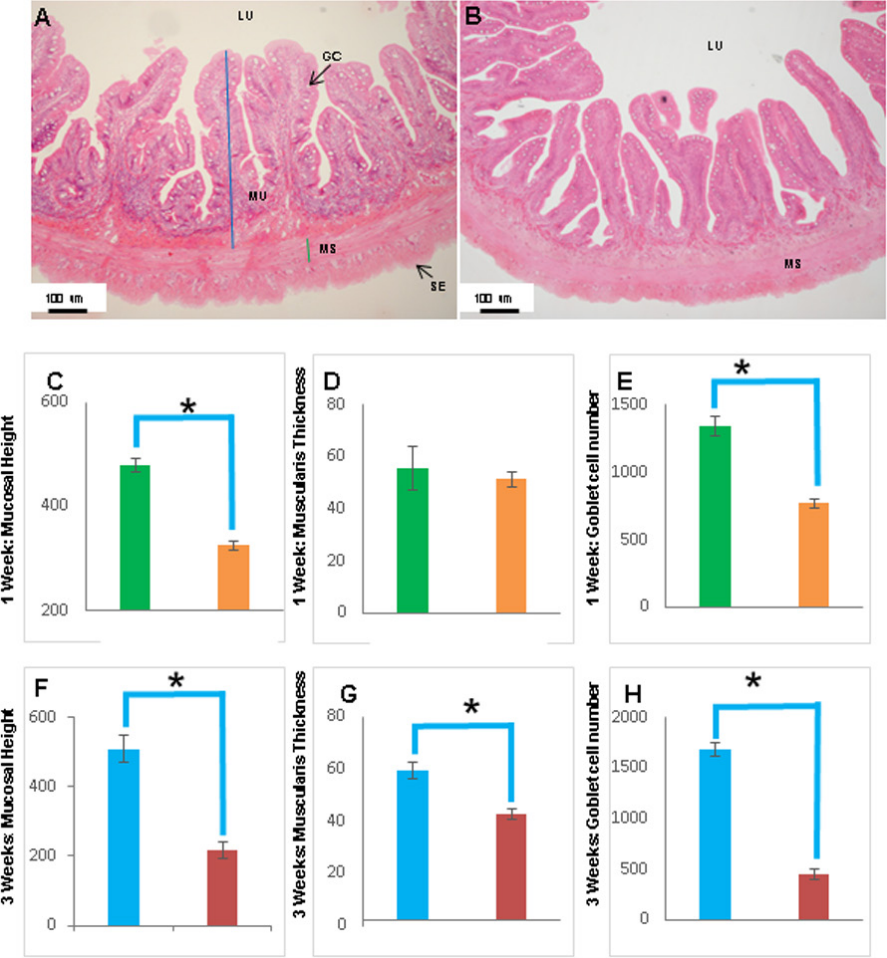
The intestinal mucosal height, muscularis thickness as well as goblet cell numbers showed significant decrease upon extended starvation. (A and B) Representative transverse section of H&E stained mid-intestine from control Asian seabass (A) and those starved for three weeks (B). Mucosal height is indicated by the blue line and muscularis thickness is indicated by the green line. Scale bar = 100 µm. (C–E) Comparison of mucosal height (C), muscularis thickness (D) and goblet cell numbers (E) between controls (green) and fish starved for one week (orange). (F–H) Comparison of mucosal height (F), muscularis thickness (G) and goblet cell numbers (H) between controls (blue) and fish starved for three-weeks (red). Significantly different parameters are indicated by ∗ (*p* < 0.05, Student’s *t*-test). Abbreviations: Serosa (SE), lumen (LU), mucosa (MU), muscularis (MS), goblet cells (GC).

The histology of the gut was also studied under the effect of two different stressors. The effect of starvation was analyzed at two different time points (one and three weeks post-starvation) (Fig. 8). It had a pronounced effect on gut histology, with a noticeable decrease in mucosal height (>30% lesser) and goblet cell numbers (>40% reduction) observed within a week of starvation (Fig. 8), whereas, the muscularis thickness showed a nominal decrease (∼4%) during this time period. The differences were much more pronounced following three weeks of starvation. The mucosal height of starved fishes was reduced by almost 60% and the goblet cells were reduced by >70% in comparison to the control fishes. In addition, the muscularis thickness also showed a significant decrease of almost 30% following three weeks of starvation (Fig. 8). Differences in salinity conditions did not have any obvious effect on the gut histology (Figs. S1 and S2).

## Discussion

The structural adaptation of the alimentary canal of each fish species is distinct and depends not only upon the body shape, weight and sex, but also upon its unique environment and the feeding behaviour (Barlow et al., 1993; Davis, 1985). Knowledge on the functional morphology of the alimentary canal would be useful for establishing a feeding regime for Asian seabass, a fish of increasing aquaculture importance, especially in South-East Asia. The Asian seabass is a carnivorous species, this feeding behaviour is reflected in its large mouth, oblique and wide upper jaw which enables the fish to swallow its prey as a whole (Pusey, Kennard & Arthington, 2004). Out of the four gill arches, the first two have thin and long rakers which are probably helpful in retaining smaller prey (Canan et al., 2012).

The Asian seabass generally feeds on prey such as molluscs, shrimps and fishes (Davis, 1985), therefore, the short and thick esophageal muscularis seems to have a protective function useful for ingestion of solid material (Domeneghini, Straini & Veggetti, 1998). The esophagus in the majority of teleost fishes is lined with stratified squamous epithelium. The closely-fitted multiple epithelial layers help to withstand various mechanical and chemical abrasions as the food enters the alimentary canal (Machado et al., 2013; Raji & Norouzi, 2010; Santos et al., 2015a; Vieira-Lopes et al., 2013). In contrast, the Asian seabass esophagus was found to be lined with simple columnar epithelium only. Another interesting feature was the presence of glands in the esophageal mucosa. Glands are not typically associated with the fish esophagus (Canan et al., 2012; Raji & Norouzi, 2010; Takiue & Akiyoshi, 2013; Xiong et al., 2011) but have been reported in a few fish species. The requirement for higher level of protection provided by the multi-layered epithelium in typical fishes could perhaps be offset by the presence of numerous mucosal glands in the Asian seabass esophagus. This is since the secretion from these glands would be useful for protecting the mucosa from mechanical wear and aid the passage of food into the stomach lining (Santos et al., 2015b; Vegas-Velez, 1972). Further, the mucous secreting cells interspersed in the simple columnar epithelium help in safeguarding the lining of the esophagus from chemical and mechanical injury and also serve as a lubricant to aid in food passage (Machado et al., 2013). In fact, the esophageal mucous cells are a feature associated with most fish species (Díaz, García & Goldemberg, 2008; Domeneghini et al., 2005; Germano et al., 2014) and could likely be serving a function equivalent to the salivary glands in mammals (Díaz, García & Goldemberg, 2008; Pedini et al., 2004). The main function of the stomach when present is the storage of food and production of hydrochloric acid to aid digestion (Gartner et al., 2002; Menin & Mimura, 1992; Stroband & Van Der Veen, 1981). The Asian seabass stomach is a straight, sac-like structure, comparable to those of other carnivorous fishes, such as pike, channel catfish and halibut (Evans & Claiborne, 2006). Though there is no clear distinction at the morphological level, histologically the stomach of the Asian seabass can be divided into cardiac, fundic and pyloric regions. This is similar to other carnivorous fish species, such as *Centropomus*, where the stomach is well defined (Machado et al., 2013). PAS staining indicated the presence of neutral glycoproteins which serve to protect the mucosal surface against microorganisms and high acidity of the stomach contents (Buddington & Doroshov, 1986; Díaz et al., 2003; Machado et al., 2013). The stomach had a large number of gastric glands, a feature typically associated with carnivorous fish species with a greater need to digest food high in protein content (Machado et al., 2013). In comparison to the other regions of the stomach, glands and surface mucus cells were scarce in the pyloric region of the stomach. This decrease is indicative of a ‘food retentive’ more than a ‘food digestive’ function for this region. The storage of food in the pyloric region (aided by the pyloric sphincter) before it enters the intestine would allow more time for digestion and has been observed in other fishes such as the walking catfish (*Clarias batrachus*) and red-bellied piranha (*Serrasalmus nattereri*; Raji & Norouzi, 2010).

Pyloric caeca are finger-like projections, highly variable in number (0–1,000 s). They are associated with the gut of the majority of fishes (∼60% of identified species) with an increased frequency seen amongst carnivorous fish species (Canan et al., 2012; Hossain & Dutta, 1996; Raji & Norouzi, 2010). Though a specific function has not been associated with these finger-like projections, the most likely option seems to be an evolutionary adaptation to facilitate the digestive process by increasing the absorptive area (Canan et al., 2012). Also, in some cases, such as the pyloric caeca of the carnivorous red-bellied piranha, an abundance of goblet cells which stain positive for neutral glycoproteins, has been observed indicating an additional role for these accessory appendages (Raji & Norouzi, 2010). In the case of Asian seabass, the five finger-like pyloric caeca have scarce neutral glycoprotein secreting mucous cells indicative of a primary absorptive function. Although evidence of endocrine activity has been shown in the pyloric caeca of some fishes (Anderson, 1986; Beorlegui, Martínez & Sesma, 1992), no secretory gland was observed in the pyloric caeca of the Asian seabass.

In teleost fishes, the length of the intestine provides a good gauge on their feeding behaviour—longer in iliophagous, omnivorous and herbivorous species, and shorter in insectivorous and carnivorous species (Canan et al., 2012; Machado et al., 2013). The intestinal coefficient of Asian seabass is relatively low (1.1) and falls within the range expected for carnivorous fishes (0.2–2.5). On the other hand, the intestinal coefficient range for omnivorous (0.6–8.0) and herbivorous (0.8–15.0) fishes are much higher (Al-Hussaini, 1949; Angelescu & Gneri, 1949; Bertin, 1958; Santos et al., 2015a; Santos et al., 2015b; Xiong et al., 2011). The Asian seabass intestine can be divided into three regions—the anterior, mid and posterior portions at the histological level, though no morphological differences existed as is the case for most fishes (Gargiulo et al., 1998; Scocco, Menghi & Ceccarelli, 1997). Numerous microvilli adorn the intestinal epithelium, which comprises of many goblet and absorptive cells to serve lubricative function as well as aid digestion and absorption of food (Gartner et al., 2002). The intestinal epithelium, similarly to the esophagus and rectum, stained positive for both acidic and neutral glycoproteins. In addition, AB staining was positive at both pH 0.5 and pH 2.5 indicating the presence of both sulphated and carboxylated glycoproteins, respectively. Although the majority of the goblet cells stained positive for both AB and PAS, some of them stained for only PAS or for AB. The co-existence of goblet cells secreting neutral or acidic+neutral glycoproteins probably represents the sequential nature of mucous biosynthesis with cells producing neutral glycoprotein only (PAS-positive) representing an earlier stage in development compared to the other cell types. On the other hand, mucins stain with AB pH 2.5 when glycoproteins are carboxylated and with AB pH 0.5–1.0 when the sulphated groups are conjugated to glycoproteins (Sarasquete et al., 2001). Additionally, the number of goblet cells increased from the anterior to posterior portions in the intestine as well as rectum. The higher number of goblet cells in the rectum seems to be a universal feature in fishes and is probably useful for increased mucous production to safeguard the intestinal lining and aid faecal expulsion (Machado et al., 2013).

In addition, gut histology was also studied upon changing the feeding and salinity conditions. Food deprivation has been documented to have a major effect on the gastrointestinal tract of several fish species such as white sturgeons (*Acipenser transmontanus*), neon damselfish (*Pomacentrus coelestis)*, Red Sea surgeonfish (*Acanthurus nigrofuscus*), common carp (*Cyprinus carpio* L.) and rainbow trout (*Oncorhynchus mykiss*). The changes reported vary from reduction in mucosal surface area, mucosal thickness and intestinal length along with decreased mucous cell numbers (Domeneghini et al., 2002; Gas & Noailliac-Depeyre, 1976; Hall & Bellwood, 1995; MacLeod, 1978; Montgomery & Pollak, 1988). A similar effect was observed on the Asian seabass gut with a pronounced decrease in all the parameters measured following three weeks of starvation. Further, the intestine of the species appears very sensitive to changes in food supply as even within a week of starvation, noticeable changes could be seen in the mucosal height as well as goblet cell numbers. This is similar to the observation in damselfish wherein 13 days of starvation resulted in a reduction of mucosal surface area, thickness and intestinal length (Hall & Bellwood, 1995). On the other hand, repeated changes in salinity did not have any obvious effect on the intestinal morpho-histology of the Asian seabass.

There are very few publications on the effect of stress on fish gut histology. In a study done on damselfishes, no apparent effect on the intestinal mucosa could be observed upon stress treatment wherein fishes were placed in tubs containing rubble (Hall & Bellwood, 1995). However, in another study, alkyl benzene sulphonate (an active ingredient found in products such as detergents, shampoos and cosmetics) had a profound effect on the gut histology with villi losing their individuality and forming a mass in young giltheads. In addition, the lamina propria couldn’t be identified and the submucosa was hypertrophied and the muscular layer was thickened in these fishes (Rosety et al., 2001). The lack of effect of salinity changes on the gut histology of Asian seabass could be attributed to the euryhaline nature of Asian seabass due to which it can adapt well to a range of salinities (Moore, 1982; Vij et al., 2014).

In conclusion, our work is the first study describing the morphological as well as histological features of the Asian seabass gut. The observations described in this publication will serve as a reference for future research aimed at studying the effect of environmental variables, stress conditions and diet on the digestive system of the species. In fact, in this study, we have already made use of this information to analyze the effect of two different stress conditions, namely changes in salinity and food deprivation, on the gut histology. Histological features of the gut were unaltered upon salinity stress, whereas starvation had a major effect on the gut in terms of reduced mucosal height, muscularis thickness and goblet cell numbers compared to control fishes. Future nutriphysiological (and possibly even nutrigenomic) studies can potentially benefit from the ‘baseline information’ described in the current publication.

##  Supplemental Information

10.7717/peerj.2377/supp-1 Data S1 Raw data for Table 2, Fig. 8 and Fig. 2

10.7717/peerj.2377/supp-2 Table S1

10.7717/peerj.2377/supp-3 Table S2 Glycoprotein histochemistry of the Asian seabass digestive tract

10.7717/peerj.2377/supp-4 Table S3

10.7717/peerj.2377/supp-5 Figure S1 Flow chart outlining the experimental setup for the salinity stress experiment. From each of the four groups (A, B, C, D), 6 mid intestine samples were collected for histological analyses. ASB: Asian seabass, dph: day post hatching.

10.7717/peerj.2377/supp-6 Figure S2 The gut histology of Asian seabass subjected to repeated salinity challenge was similar to those of controls. The fishes were subjected to a simple salinity challenge through a transfer from full seawater (30–32 ppt salinity) to freshwater (0 ppt) and grown for three weeks before sampling, whereas the controls were grown in seawater all along (A–E). Representative transverse section of H&E stained mid-intestine from control (A) and sample (B) Asian seabass. Comparison of mucosal height (C), muscularis thickness (D) and goblet cell numbers (E) between control (green) and Asian seabass subject to salinity conditions (orange). The fishes were also subjected to repeated salinity challenge through a transfer from full seawater (30–32 ppt salinity) to freshwater (0 ppt), keeping them there for three weeks and transferring them back to seawater and grown for three weeks before sampling, whereas the controls were grown in seawater all along (F–J). Representative transverse section of H&E stained mid-intestine from control (F) and sample (G) (transferred from seawater to fresh water (grown for three weeks) and again to seawater (grown for three weeks) before sampling) Asian seabass. Mucosal height is indicated by blue line and muscularis thickness is indicated by green line. Scale bar =2000*B*5m. Comparison of mucosal height (H), muscularis thickness (I) and goblet cell numbers (J) between control (green) and Asian seabass subject to salinity conditions (orange). Serosa (SE), lumen (LU), mucosa (MU), muscularis (MS), goblet cells (GC).

## Additional Information and Declarations

### Data Availability

The following information was supplied regarding data availability: The raw data has been supplied as Data S1.
