# Supplementary figures and images for: Morpho-histological characterisation of the alimentary canal of an important food fish, Asian seabass (Lates calcarifer)
Source: PeerJ. 2016 Aug 24;4:e2377. doi: 10.7717/peerj.2377 (PMC5012279; doi:10.7717/peerj.2377)

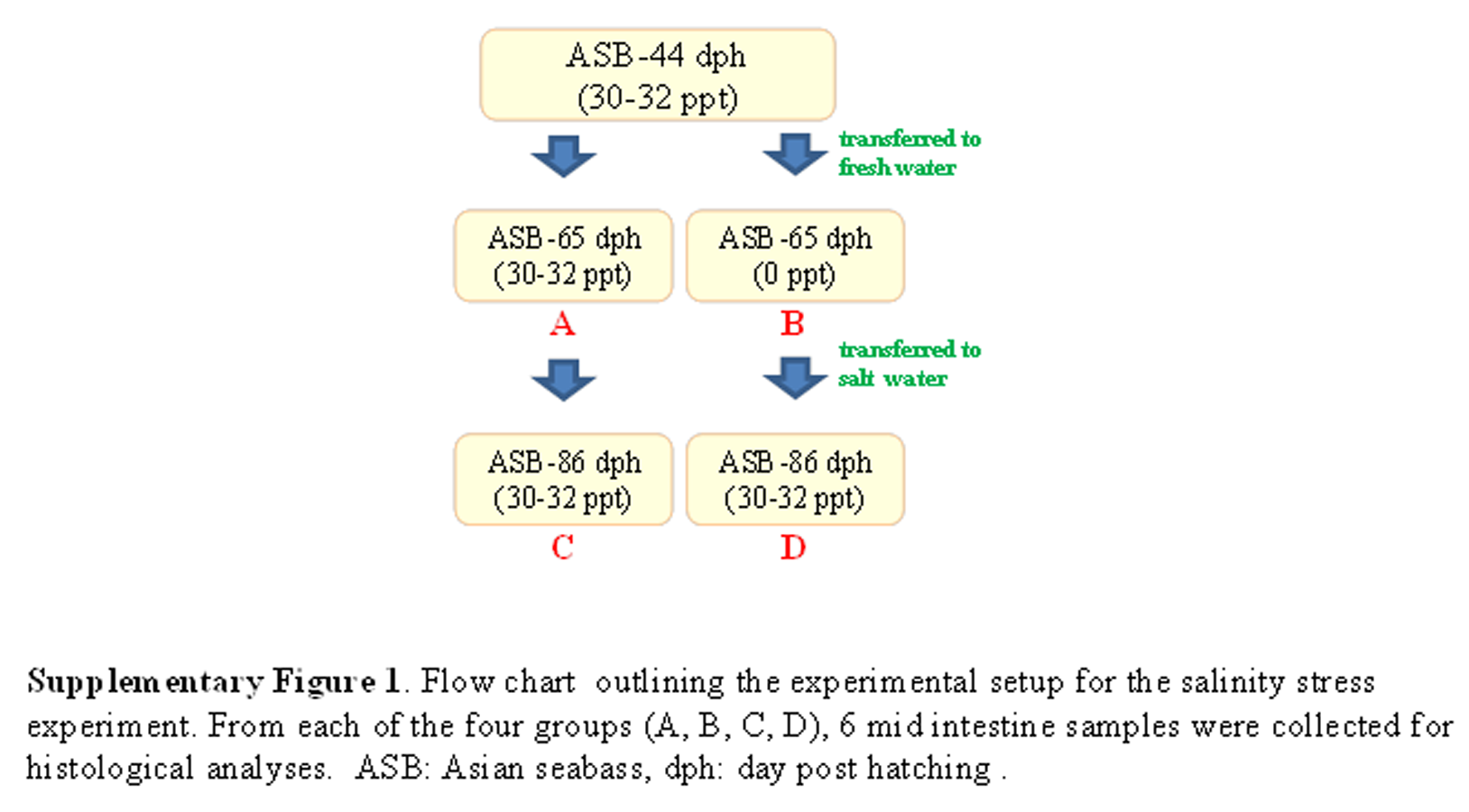

Supplement: Figure S1 — From each of the four groups (A, B, C, D), 6 mid intestine samples were collected for histological analyses. ASB: Asian seabass, dph: day post hatching. [file peerj-04-2377-s005.png]

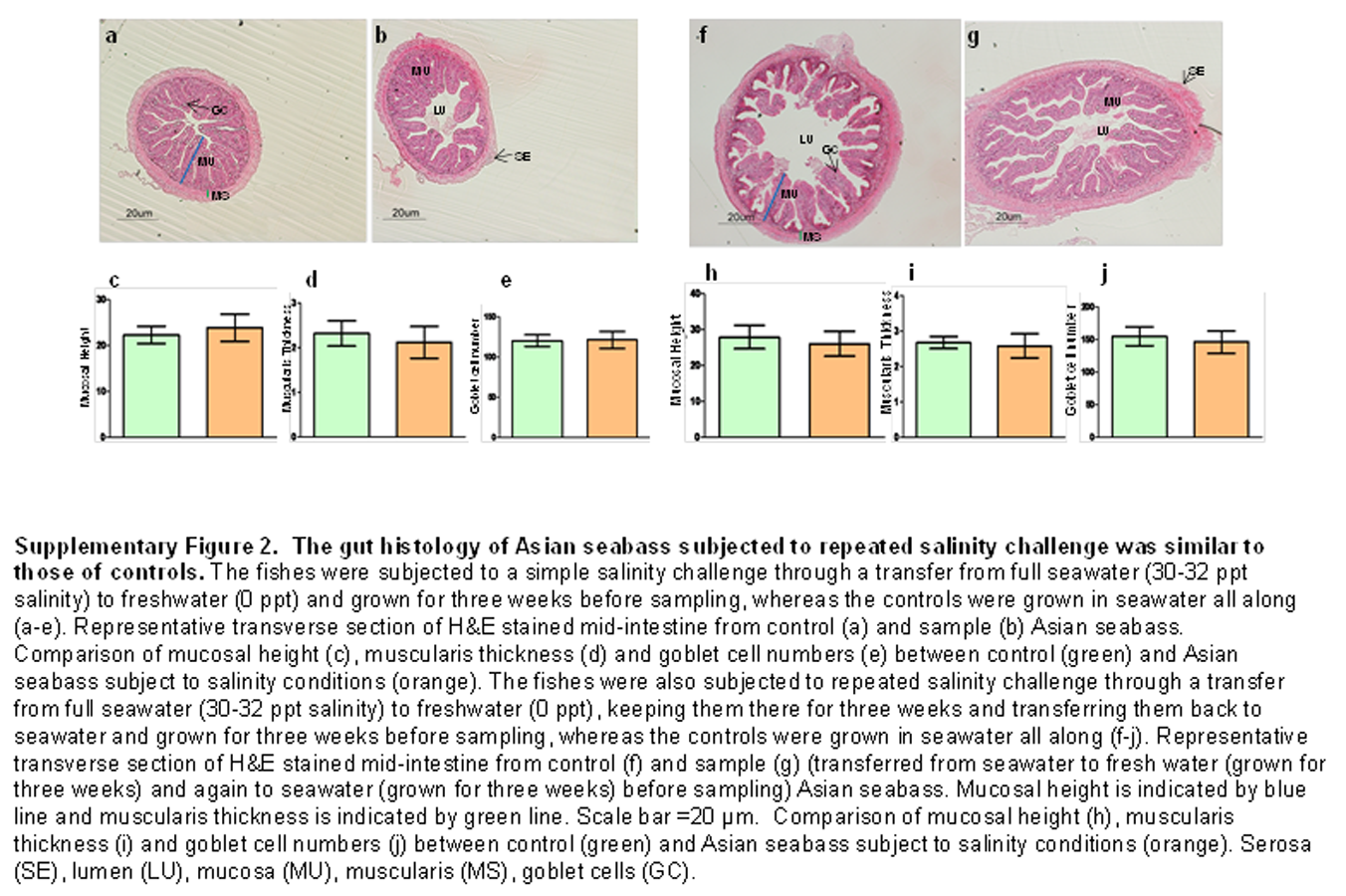

Supplement: Figure S2 — The fishes were subjected to a simple salinity challenge through a transfer from full seawater (30–32 ppt salinity) to freshwater (0 ppt) and grown for three weeks before sampling, whereas the controls were grown in seawater all along (A–E). Representative transverse section of H&E stained mid-intestine from control (A) and sample (B) Asian seabass. Comparison of mucosal height (C), muscularis thickness (D) and goblet cell numbers (E) between control (green) and Asian seabass subject to salinity conditions (orange). The fishes were also subjected to repeated salinity challenge through a transfer from full seawater (30–32 ppt salinity) to freshwater (0 ppt), keeping them there for three weeks and transferring them back to seawater and grown for three weeks before sampling, whereas the controls were grown in seawater all along (F–J). Representative transverse section of H&E stained mid-intestine from control (F) and sample (G) (transferred from seawater to fresh water (grown for three weeks) and again to seawater (grown for three weeks) before sampling) Asian seabass. Mucosal height is indicated by blue line and muscularis thickness is indicated by green line. Scale bar =2000B5m. Comparison of mucosal height (H), muscularis thickness (I) and goblet cell numbers (J) between control (green) and Asian seabass subject to salinity conditions (orange). Serosa (SE), lumen (LU), mucosa (MU), muscularis (MS), goblet cells (GC). [file peerj-04-2377-s006.png]
